# Immunopathogenesis and Novel Therapeutics Strategies of Systemic Lupus Erythematosus

**DOI:** 10.3390/ijms24119470

**Published:** 2023-05-30

**Authors:** Przemysław Kotyla, Marzena Olesińska

**Affiliations:** 1 Department of Internal Medicine, Rheumatology and Clinical Immunology, Faculty in Katowice, Medical University of Silesia, 40-635 Katowice, Poland; 2 Department of Connective Tissue Diseases, National Institute of Geriatrics, Rheumatology and Rehabilitation, 02-637 Warsaw, Poland

Systemic lupus erythematosus is a chronic connective tissue disease of unknown origin and unpredictable course. It commonly serves as a prototype for almost all connective tissue diseases and more general autoimmune diseases. In the course of the disease, almost all vitally important body organs may be affected; thus, SLE is considered a disease with “many faces”. In line with this implication, various pathophysiological mechanisms might be involved in internal organ damage.

Systemic lupus erythematosus still garners high scientific interest. Clinicaltrials.gov registry encompasses 838 clinical trials currently ongoing in SLE. Unfortunately, for many years, this scientific interest has not been translated to the development of new therapeutic strategies. In the last 20 years, only two new molecules were introduced into the therapeutic repertoire in lupus (belimumab and recently anifrolumab).

This Special Issue entitled “Immunopathogenesis and Novel Therapeutics Strategies of Systemic Lupus Erythematosus” highlights the recent progress in the field of pharmacology and pathomechanism of the disease.

SLE is a disease affecting preferably young, childbearing women. This leads to many therapeutic consequences, the most important of which is effective contraception. In this issue, Juif et al. investigated the effect of cenerimod, a selective S1P1 receptor modulator, on the pharmacokinetics of a combined oral contraceptive.

The activation of the S1P1 receptor is involved in the egress of lymphocytes from secondary lymphoid organs into the vascular circulation; thus, the inhibition of the receptor prevents the movement of lymphocytes from lymphoid organs to sites of inflammation. Recently, cenerimod, an S1P1 receptor modulator, is under investigation for its use in the treatment of SLE [1]. Taking into account the high teratogenicity of the drug, it can be administered only when highly effective contraception is used. The main question is whether concomitant treatment with cenerimod and oral contraceptives may influence the metabolism of oral contraceptives and, in some instances, reduces the effectiveness of contraceptives. According to the results of the study by Juif et al., the administration of combined oral contraceptives alone or together with cenerimod was safe and well tolerated. This may open the way for further studies with cenerimod in young women with lupus, who wish to or have to use highly effective contraception.

Pulmonary arterial hypertension (PAH) is still an unmet need in the treatment of SLE. Moreover, PAH in SLE patients dramatically complicates the course of the disease, contributing to premature death in this group of patients [2]. In this issue of the *International Journal of Molecular Sciences*, Yen and colleagues used an animal model of SLE- associated PAH to test the role of Cathepsin S (Cat S) inhibitors in treating SLE-associated PAH. It was recently established that Cat S overexpression is responsible for pulmonary arterial remodeling, directly leading to the development of PAH [3]. According to the results from the study, the Cat S inhibitor Millipore-219393 was found to be an effective treatment in an established experimental model of SLE associated with PAH. The treatment with this Cat S inhibitor reduced right ventricular hypertrophy and pulmonary arterial remodeling in female MRL/lpr (SLE) mice. In the second part of the study, the authors revealed that Cat S inhibitors upregulate PPARγ protein levels, which translates to a reduction in Cat S expression. We are still far away to understand all autoimmune phenomena that may at least partially explain the complicated clinical picture of SLE. Taking into account the role of autoantibodies generated in the course of the disease, it is reasonable to seek abnormalities in the function of B cells. This was the background of the study of Szabo et al. [4], who aimed to analyze the distribution of the circulating follicular T-helper (cT_FH_) and T-regulatory (cT_FR_) cells and their association with disease characteristics and B-cell composition in SLE. They showed a higher frequency of cT_FH_ and cT_FR_ in cutaneous lupus and a reduced frequency of cT_FH_ 17 in lupus nephritis patients. They also confirmed the role of Il21, mainly produced by T_FH_, in orchestrating the plasmablast generation and autoantibody production. 

Another approach to restoring the imbalance between various Th-cell populations is presented by Kuca-Warnawin et al. [5]. The authors focused on the properties of mesenchymal stem cells, pluripotential cells, able to influence the Th subset differentiation in two autoimmune diseases SLE and systemic sclerosis. In detail, the mixture of adipose-derived mesenchymal cells and Cd4^+^ cells modulates T-cell differentiation toward Th2 response with a concomitant reduction in IFNγ/Il-4 ratio, promoting Th17 formation and Il-17 synthesis and upregulate classical Treg formation. At the moment, the pathophysiological role of these findings should be elucidated, and perhaps the clinical importance of the results depends on the immunological mechanisms of a given disease (Th1 predominance in systemic sclerosis versus Th2 response in SLE).

There is growing evidence on the role of interferon activity in the development and progression of SLE, and dysregulated type I interferon signaling is extensively reported in SLE as well as in other autoimmune diseases [6]. In their review, Infante et al. [7] focused on the transduction pathways of type I interferons, in particular INFα and its immune regulatory function in the pathogenesis of SLE, with a special emphasis on lupus nephritis. The authors extensively discussed the mutual interaction between various immunocompetent cells able to synthesize or regulate the synthesis of IFN α. Additionally, they highlighted the role of IFN in kidney damage in the course of lupus nephritis. They pointed to several mechanisms of kidney damage in which IFN played a role, such as proinflammatory cytokine production, direct podocyte damage, and IFN-regulated leukocyte infiltration of kidney parenchyma. The authors also reviewed the role of the NF-kB pathway in proinflammatory signaling, which mediates glomerular and tubulointerstitial damage in LN patients. Taking into account the activity of IFN on NF-kB hyperactivation, renal damage may be at least partially mediated by IFN, and this finding may be translated into the use of anti-IFN antibodies to stop LN.

The last study included in this issue was conducted at an ophthalmological center in Poland, one of the best-known ophthalmological centers in this part of Europe specializing in the treatment of ocular diseases among patients with connective tissue diseases. In their paper, Luboń et al. [8] discussed in detail all the ophthalmological aspects of SLE, with special emphasis on the clinical picture and possible autoimmune mechanisms responsible for ocular presentation in lupus patients. The involvement of eye mechanisms was underlined many years ago considering the role of ocular implantation in the most commonly used disease activity index—BILAG. Even though this excellent tool collects data on several aspects of eye involvement, awareness of eye involvement in the rheumatological community is very limited. We do hope that paper of Luboń et al. shed new light on this problem.
